# A blood-based mRNA signature distinguishes people with Long COVID from recovered individuals

**DOI:** 10.3389/fimmu.2024.1450853

**Published:** 2024-12-03

**Authors:** Daniel Missailidis, Esmaeil Ebrahimie, Manijeh Mohammadi Dehcheshmeh, Claire Allan, Oana Sanislav, Paul Fisher, Stephanie Gras, Sarah J. Annesley

**Affiliations:** ^1^ Department of Microbiology, Anatomy, Physiology and Pharmacology, La Trobe University, Bundoora, VIC, Australia; ^2^ Genomics Research Platform, School of Agriculture, Biomedicine and Environment, La Trobe University, Bundoora, VIC, Australia; ^3^ School of Animal and Veterinary Sciences, Faculty of Sciences, Engineering and Technology, University of Adelaide, Adelaide, SA, Australia; ^4^ Infection & Immunity Program, La Trobe Institute for Molecular Science (LIMS), La Trobe University, Bundoora, VIC, Australia; ^5^ Department of Biochemistry and Chemistry, School of Agriculture, Biomedicine and Environment, La Trobe University, Bundoora, VIC, Australia; ^6^ Department of Biochemistry and Molecular Biology, Monash University, Clayton, VIC, Australia

**Keywords:** COVID-19, Long COVID, biomarker, transcriptomics, inflammation, LILRB1, LILRB2

## Abstract

**Introduction:**

Long COVID is a debilitating condition that lasts for more than three months post-infection by SARS–CoV–2. On average, one in ten individuals infected with SARS CoV- 2 develops Long COVID worldwide. A knowledge gap exists in our understanding of the mechanisms, genetic risk factors, and biomarkers that could be associated with Long COVID.

**Methods:**

In this pilot study we used RNA-Seq to quantify the transcriptomes of peripheral blood mononuclear cells isolated from COVID-recovered individuals, seven with and seven without Long COVID symptoms (age- and sex-matched individuals), on average 6 months after infection.

**Results:**

Seventy genes were identified as significantly up- or down-regulated in Long COVID samples, and the vast majority were downregulated. The most significantly up- or downregulated genes fell into two main categories, either associated with cell survival or with inflammation. This included genes such as *ICOS* (FDR p = 0.024) and *S1PR1* (FDR p = 0.019) that were both up-regulated, indicating that a pro-inflammatory state is sustained in Long COVID PBMCs compared with COVID recovered PBMCs. Functional enrichment analysis identified that immune-related functions were expectedly predominant among the up- or down-regulated genes. The most frequently downregulated genes in significantly altered functional categories were two leukocyte immunoglobulin like receptors *LILRB1* (FDR p = 0.005) and *LILRB2* (FDR p = 0.027). PCA analysis demonstrated that *LILRB1* and *LILRB2* expression discriminated all of the Long COVID samples from COVID recovered samples.

**Discussion:**

Downregulation of these inhibitory receptors similarly indicates a sustained pro-inflammatory state in Long COVID PBMCs. *LILRB1* and *LILRB2* should be validated as prospective biomarkers of Long COVID in larger cohorts, over time and against clinically overlapping conditions.

## 1 Introduction

Millions of individuals are suffering from ongoing or new symptoms weeks, months or years after initial infection with SARS-CoV-2 (1). This condition is most often called Long COVID, also referred to as post-acute sequelae of SARS-CoV-2 infection (2). Many studies have now been conducted to measure the prevalence rates of Long COVID, varying widely from 9-83% of people who had been infected with SARS-CoV-2 (3–5). This large variation can be attributed to differences in clinical definition, methodology, vaccination status and the severity of acute infection. Conservative estimates suggest that one in ten people previously infected with SARS-CoV-2 have developed Long COVID (6). Long COVID has been defined by the World Health Organisation (WHO) as the occurrence of new or persistent symptoms three months after a SARS-CoV-2 infection, which persists for at least two months and cannot be explained by an alternative diagnosis (7). There have been over two-hundred symptoms reported which affect most body systems (8). The most common symptoms reported are fatigue (prevalence rate of 21.6%), respiratory problems (14.9%), cognitive impairment (10.1%) and joint/muscle pain (10.6%) (8). The underlying molecular mechanisms responsible for progression to chronicity following the initial infection are not known. Several theories focusing on immune dysfunction have been proposed, relating to viral persistence, reactivation of latent viruses, increased production of autoantibodies and persistent inflammation (6).

Despite the high occurrence rate of Long COVID there are no identified risk loci, diagnostic tests, treatments, or specific clinical biomarkers. This adds a layer of subjectivity and exclusionary process to Long COVID diagnosis that may delay or confound effective clinical management. It also poses a challenge in the recruitment of stringently diagnosed cohorts for research. Considering these issues, many studies have been undertaken which seek biomarkers of Long COVID. These efforts have mostly focused on attempting to identify immunological, neurological, vascular and cardiac signatures specific to the disease. Several studies have reported circulating inflammatory marker proteins to associate with Long COVID disease status, with this body of work strongly indicating a sustained pro-inflammatory state in at least a subset of affected individuals (9–16).

One study examined the immunological profile of Long COVID-affected individuals compared to COVID recovered individuals with varying severity of acute illness. Long COVID-affected individuals were characterized by increased IFN-γ and IL-2, indicative of a pro-inflammatory state and decreased CCL4 production. The authors propose that while the increased IFN-γ and IL-2 results in activation of effector T cells, the concomitantly reduced CCL4 leads to impaired recruitment of them to infected sites (17).

An Australian longitudinal study investigated the transcriptional and immunological blood profile of sixty-nine COVID recovered patients of varying severities, including twenty-one individuals referred to a Long COVID clinic (18). The study found that transcriptional changes which occurred during acute infection were still evident in recovered individuals for at least six months post-infection. This highlights the importance of including COVID recovered samples as controls in new studies. Crucially this study also reported that enriched immune signatures identified in whole blood transcriptomics withstood correction against the differential proportions of cell types in each PBMC sample, thereby demonstrating that transcriptomic assessment of heterogeneous PBMCs in COVID recovered samples is robust against individualized changes in cell type proportions and thus can be applied with confidence in future studies.

Measuring levels of specific mRNA transcripts from blood has high diagnostic potential since blood is readily accessible and the transcripts are measurable by existing Real-Time Quantitative Reverse Transcription PCR (qRT-PCR) infrastructure. This benefit would be accentuated if the number of transcripts required to indicate disease status is few. PBMCs are very easily isolated from whole blood and since, taken together, the prior studies strongly suggest persistent immunological disturbances (including robust changes to PBMCs), PBMC mRNA transcripts as potential discriminators of disease status are a promising avenue.

With this in mind, we undertook a small pilot study to test the effectiveness of using differences in PBMC gene expression to identify candidate biomarkers of Long COVID. We used RNA-Seq to sequence and quantify the transcriptomes of PBMCs isolated from seven Long COVID and seven COVID recovered age and sex matched individuals. The Long COVID participants had been infected with SARS-CoV-2 on average six months prior to sample collection. Functional enrichment analysis identified that immune-related functions were unsurprisingly predominant among the up- and down-regulated genes. The differentially expressed genes supported a persistent or chronic inflammatory state in Long COVID. Using multivariate-based signature discovery, we identified a robust blood-based transcriptomic signature that effectively distinguished patients who have fully recovered from SARS-CoV-2 (COVID recovered) from those experiencing Long COVID using the reduced expression levels of only two genes (*LILRB1* and *LILRB2*). These results if validated in a larger cohort, over time and against similar conditions show promise for development into a simple blood based diagnostic tool for Long COVID.

## 2 Materials and methods

### 2.1 Recruitment, sampling and cohort characteristics

All participants were recruited in accordance with La Trobe University Human Ethics approvals HEC21207 and HEC21907. Seven Long COVID and seven COVID recovered participants were recruited for the study and all participants provided informed consent. Long COVID participants were defined according to the WHO description as exhibiting new or persistent symptoms three months following SARS-CoV-2 infection. Participants were asked to complete a symptom questionnaire and rate their illness severity using a five-point Likert scale. The cohort characteristics are shown in **Table 1**. COVID recovered participants were age- and sex-matched to the Long COVID cohort and on average all participants in this study were recruited approximately 6 months after an acute SARS-CoV2 infection. To exclude known, measurable alternative explanations for fatigue, blood samples collected from Long COVID participants were subject to pathology analysis conducted by Dorevitch Pathology, Melbourne, Australia. Blood samples for subsequent PBMC isolation were collected in lithium-heparin tubes.

**Table 1:** Participant cohort information.

Participant information	Long COVID	COVID recovered
Total Participants	7	7
Average age (years)	41	45
Age range (years)	23-53	23-63
Female (%)	85.7	71.4
Male (%)	14.3	28.6
Average time post-COVID (months)	6.1	5.5
Hospitalisation (number of participants)	1	n/a

### 2.2 PBMC isolation

PBMCs were isolated as previously published (19). Briefly, whole blood was separated by centrifugation in SepMate tubes (STEMCELL Technologies) at 1200×G for 10 minutes at RT. Resultant PBMCs were washed with RPMI 1640 to remove residual Ficoll. Aliquots were frozen in fetal bovine serum with 10% DMSO, gradually at -80°C.

### 2.3 RNA extraction and RNA-seq

RNA was extracted from PBMCs using the Monarch Total RNA Miniprep Kit (NEB, Ipswich, MA, USA). Approximately 5 × 10^6^ PBMCs were harvested by centrifugation at 500 × g for 5 min and resuspended in 300 µL of RNA lysis buffer. The sample was transferred to a gDNA removal column fitted to a microcentrifuge tube and spun at maximum speed for 30 s to collect any contaminating genomic DNA. The RNA in the flow-through was precipitated by the addition of 300 µL ethanol (Chem Supply, Port Adelaide, SA, Australia) and mixed by repeated pipetting. The suspension was transferred to an RNA purification column fitted to a collection tube and again spun at max speed for 30 s and the flow-through discarded. The RNA was washed by the addition of 500 µL RNA wash buffer and centrifugation, as described previously, and the flow-through was discarded. The sample was DNase-treated by the addition of 5 µL DNase I and 75 µL of DNase I reaction buffer to the column matrix and incubated at RT for 15 min. A 500 µL volume of RNA priming buffer was added to the column, centrifuged for 30 s at max speed, and the flow-through was discarded. The RNA in the column was washed twice with 500 µL of RNA wash buffer, followed by centrifugation as described previously. RNA was eluted by the addition of 50 µL nuclease-free water and centrifugation at max speed for 30 s. RNA was then stored at -80°C before being sent to Novogene, Singapore on dry ice for mRNA sequencing and quantification using the Illumina NovaSeq 6000 platform and paired-end 150 bp reads.

### 2.4 Transcriptomic data analysis, enrichment analysis, and signature discovery

Transcriptomic profiling of PBMC samples from RNA-Seq Long COVID patients (n = 7) and COVID recovered individuals (n = 7) was performed by RNA-Seq (Illumina, paired end reads, 150bp). Quality control of the generated reads, mapping, and selection of genes with significant differential expression was carried out using CLC Genomics Workbench package 22 (QIAGEN) (20), HiSAT2 (21), and edgeR (22). Human genome 38 and its annotations (GRCh38) were downloaded from Ensembl genome browser (https://www.ensembl.org/index.html) and used for mapping and expression profiling. Mapping was carried out with the following parameters: mismatch cost of 2, insertion cost of 3, deletion cost of 3, minimum length fraction of 0.8, and minimum similarity fraction = 0.8. “Reads Per Kilobase of transcript per Million mapped reads” (RPKM) was used as the expression measurement. We used Generalized Linear Models (GLM) based on Negative Binomial distribution for differential expression analysis (22). The p-values were also corrected with false discovery rate (FDR) for multiple testing, and pFDR = 0.05 was used for selection of genes with statistically significant differential expression. The advantage of GLM is fitting the curve to expression values without the assumption that the error is normally distributed. Genes with an average RPKM lower than 4 in both cohorts were removed from the list of significant differentially expressed genes.

To find the key functions that the differentially expressed genes were involved in, enrichment analysis of Gene Ontology (GO) terms was analysed using the STRING web application tool (https://string-db.org/) (23). The significant molecular functions were selected based on pFDR = 0.05 and strength of the function, calculated by STRING (23).

Multivariate analysis, including principal component analysis (PCA) using correlation matrix and hierarchical clustering using average linkage method, was performed on expression values as previously described (24). PCA and clustering were utilized to evaluate the power of the developed transcriptomic signature as well as the functional pathways in distinguishing Long COVID from COVID recovered samples. Minitab Statistical Software 21 was employed for performing multivariate analysis.

## 3 Results

### 3.1 Long COVID participant symptoms

**Figure 1:**
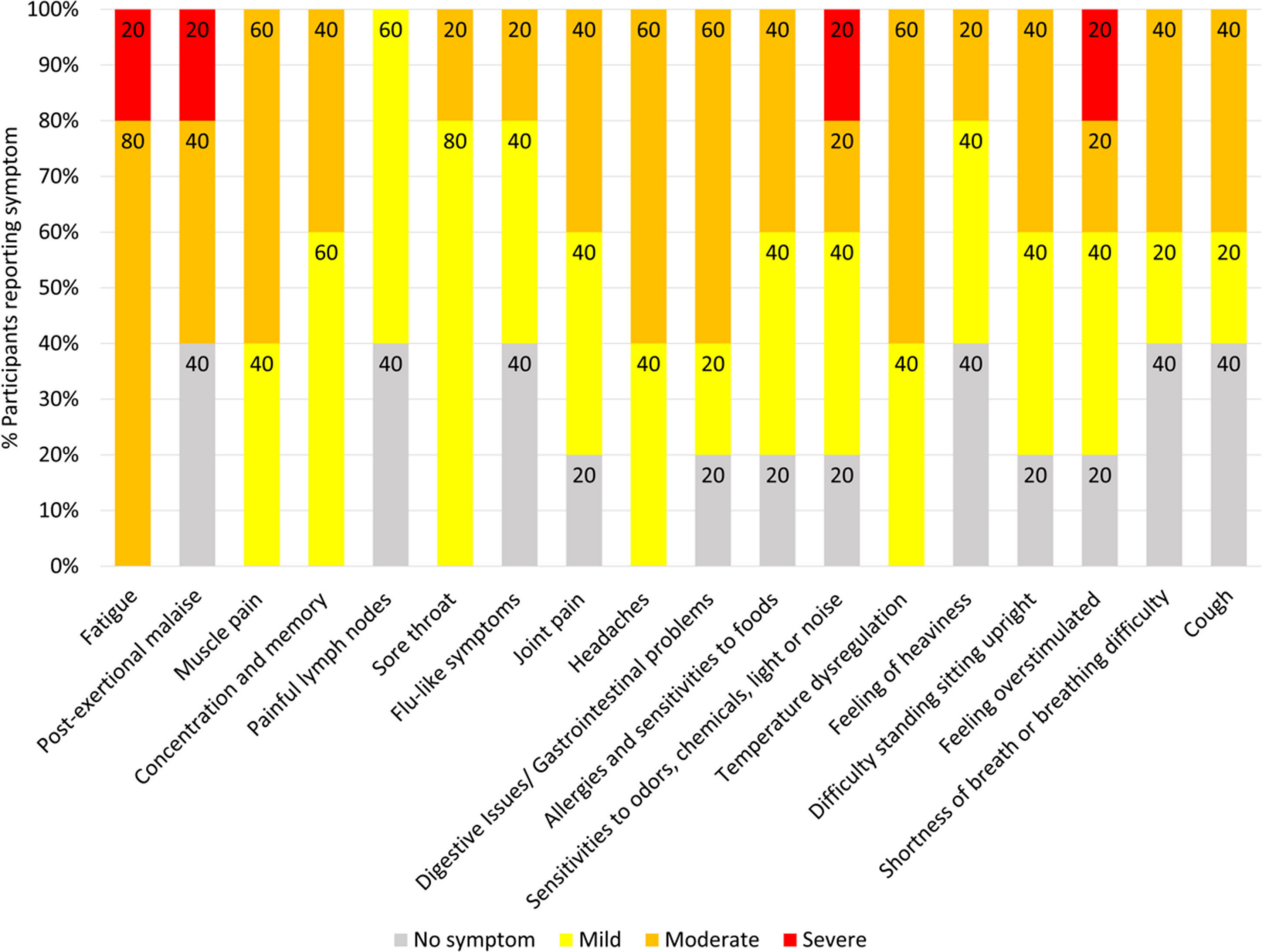
Symptoms affecting Long COVID participants and severity ratings. Seven of the participants with Long COVID completed questionnaires to list and rate the severity of their symptoms. Ratings of the symptoms were classified as 0 = no symptoms, 1-2 = mild, 3-4 = moderate, 5 = severe. Percentages are rounded to the nearest single decimal place.

Seven Long COVID and seven COVID recovered participants were included in this study (**Table 1**). All participants had reported a SARS-CoV-2 infection on average six months prior to sample collection and pathology tests revealed no underlying conditions typically associated with fatigue. All pathology results were within the normal range. There were no significant difference in the mean age of each clinical group (p = 0.58, independent t-test) or gender, but both had a higher percentage of women and middle-aged participants. This is in line with the current literature that indicates a high prevalence in this age group and in women (25). Each of the seven Long COVID participants completed the symptom severity questionnaires which used a five-point Likert scale. Eighteen symptoms were included in the questionnaire and encompassed the main symptom clusters of neurocognitive, airway, cardiopulmonary, musculoskeletal and gastrointestinal issues. The most reported symptoms were fatigue, post-exertional malaise (PEM), muscle pain, concentration and memory issues, sore throat, headaches and temperature dysregulation. Fatigue was reported by each of the Long COVID participants and five out of the seven reported PEM. Fatigue and PEM were rated with the greatest overall severity of all symptoms (**Figure 1**).

### 3.2 Transcriptomic signature of long COVID

**Figure 2:**
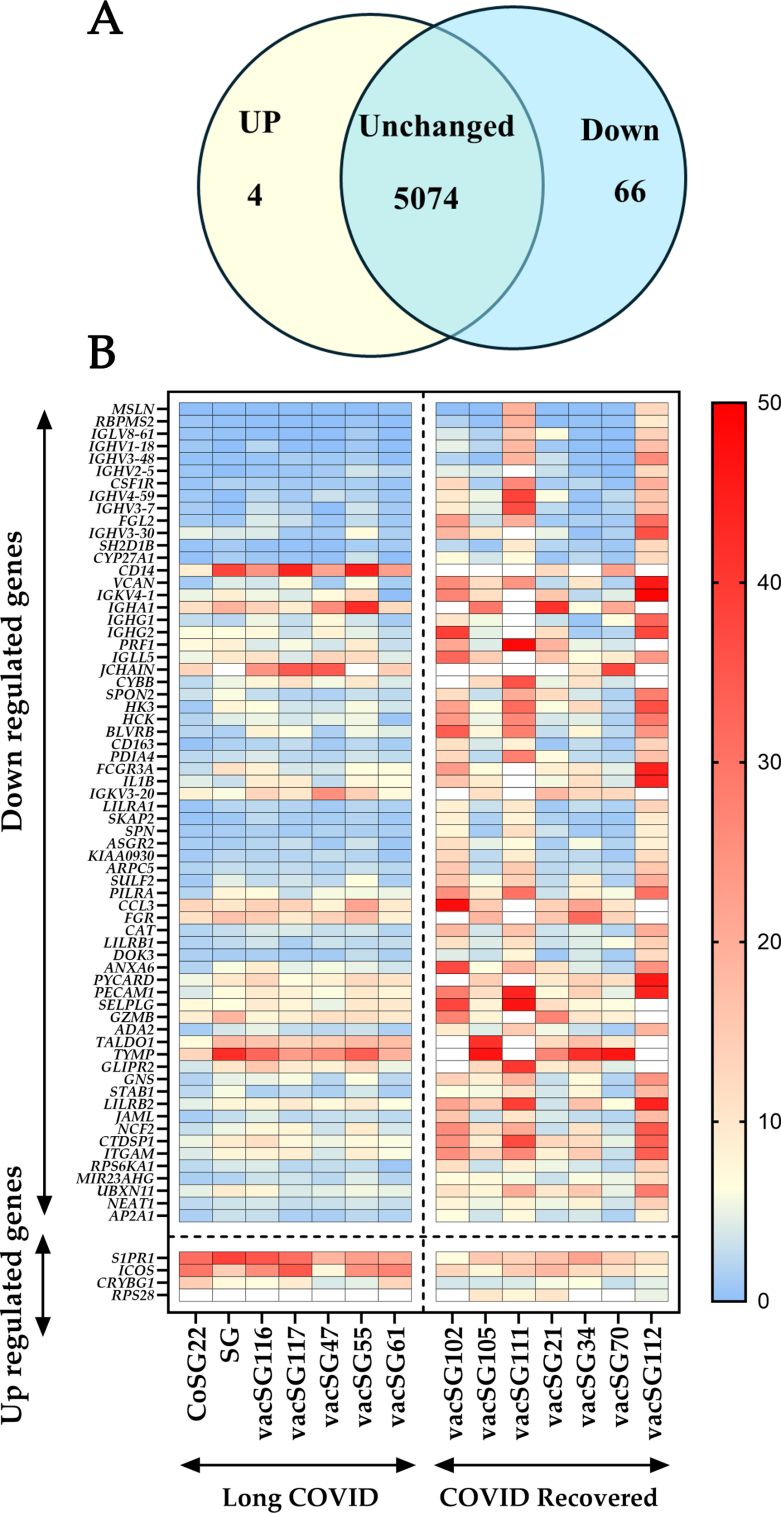
Long COVID versus COVID recovered PBMC transcriptomes. **(A)** Venn diagram depicting the number of gene transcripts significantly up- or down-regulated in PBMCs from people with Long COVID versus PBMCs from COVID recovered individuals. The p-values which determined significance were corrected with FDR for multiple testing, (pFDR < 0.05 was used as the threshold for significance). Genes with an average reads per kilobase per million mapped reads (RPKM) lower than four in both cohorts were removed from the list of significant differentially expressed genes. This was to ensure that any identified genes were present in sufficient amounts to be detected via clinically appropriate methods. **(B)** Heatmap representation of seventy differentially expressed genes between Long COVID and COVID recovered PBMC samples. Red indicates higher expression while blue indicates lower expression. Each column is a different sample and each row is a different gene.

Total RNA was isolated from PBMCs of all participants and sent for RNA-Seq analysis (26). Transcriptomic analysis identified 5,144 transcripts, of which seventy were up- or down-regulated in Long COVID compared to COVID recovered controls (**Figure 2**). Most of the differentially expressed transcripts were downregulated (sixty-six down in total) and four were signficantly up-regulated in Long COVID compared to the COVID recovered controls (FDR p value < 0.05).

**Figure 3:**
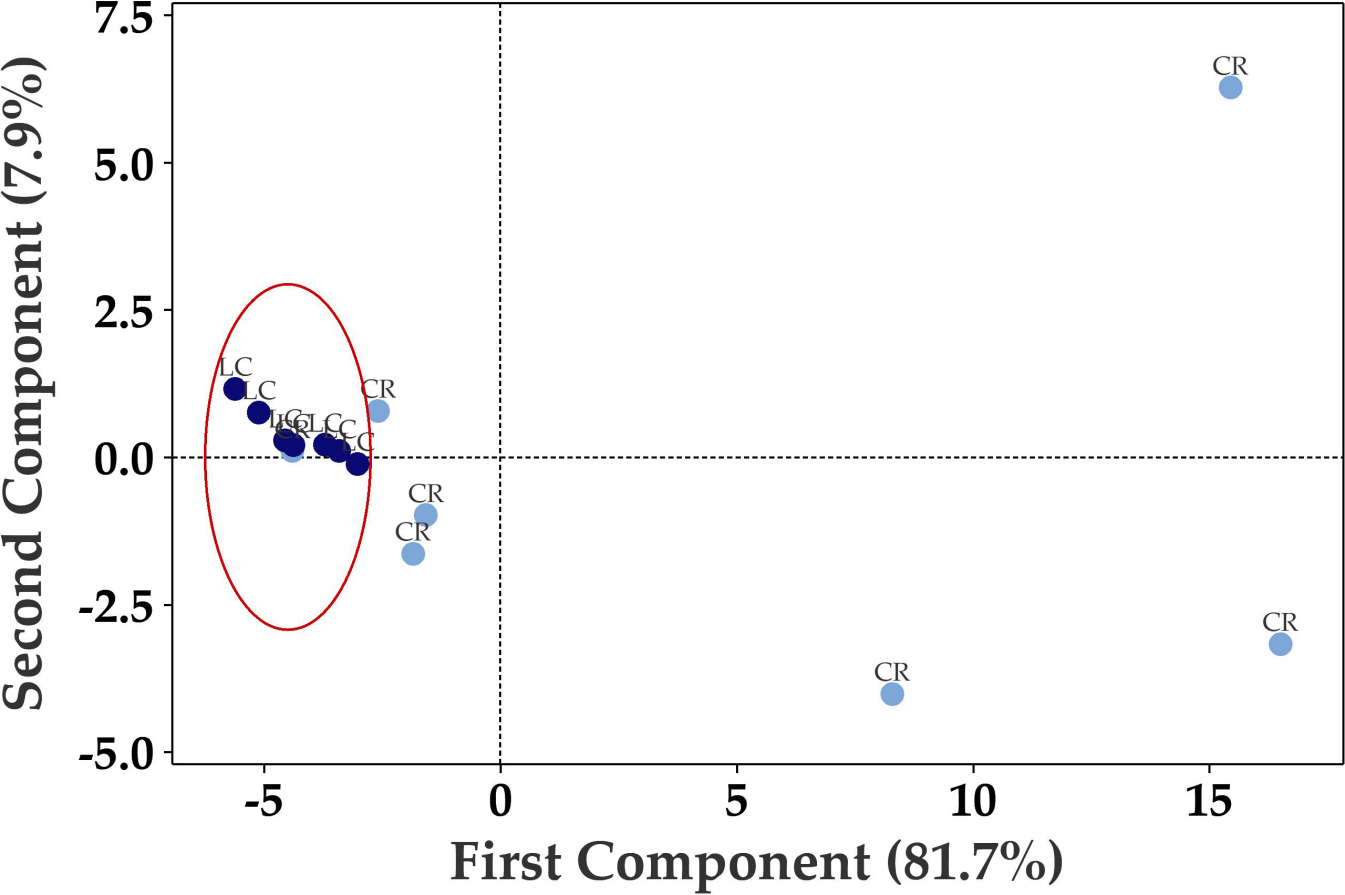
Principal component analysis (PCA) plot based on 70 differentially expressed genes in PBMCs efficiently distinguishes Long COVID (LC, red) PBMCs from most of the COVID recovered PBMCs (CR, blue) with one overlapping COVID recovered sample.

Principal Component Analysis (PCA) of the seventy up- or down-regulated genes showed separation between Long COVID and COVID recovered samples (**Figure 3**) where only one COVID recovered sample was clustered in the Long COVID group. The first component was used to classify the samples and explained 81.7% of the variation in the expression data.

### 3.3 Dysregulation of immune related pathways

The list of genes with significant (pFDR < 0.05) differential expression between Long COVID and COVID recovered PBMC samples is presented in **Table 2**. Among the top ten downregulated genes in Long COVID as determined by fold change and FDR corrected p-value were genes associated with cell survival. This included Mesothelin (*MSLN*), expression of which was reduced more than sixty-fold. Mesothelin is a cell surface glycoprotein and is involved in cell signalling and adhesion. It is associated with various cancers and is thought to promote proliferation through activation of the NF-kB pathway. Downregulation of this gene has been connected with increased cell death via apoptosis (27). Another gene in the top ten which was associated with cell survival is colony stimulating factor receptor (CSF1R). This has been shown to be important for the survival, differentiation and proliferation of myeloid cells. The RNA binding protein, mRNA processing factor 2 (RBPMS2) was also highly downregulated and functions in post-transcriptional regulation of gene expression. Inactivation of this gene via methylation has also been associated with increased apoptosis (28).

**Table 2:** The blood-derived PBMC transcriptomic signature of Long COVID compared to COVID recovered individuals The top genes were selected by satisfying a FDR p-value < 0.05 threshold.

No	Name	Chromosome	Identifier	Fold change	FDR p-value	Long COVID
1	*MSLN*	16	ENSG00000102854	-60.251	0.005	DOWN
2	*RBPMS2*	15	ENSG00000166831	-17.944	0.005	DOWN
3	*IGLV8-61*	22	ENSG00000211638	-16.350	0.005	DOWN
4	*IGHV1-18*	14	ENSG00000211945	-9.798	0.047	DOWN
5	*IGHV3-48*	14	ENSG00000211964	-8.956	0.027	DOWN
6	*IGHV2-5*	14	ENSG00000211937	-8.055	0.031	DOWN
7	*CSF1R*	5	ENSG00000182578	-7.028	0.000	DOWN
8	*IGHV4-59*	14	ENSG00000224373	-6.375	0.009	DOWN
9	*IGHV3-7*	14	ENSG00000211938	-5.779	0.032	DOWN
10	*FGL2*	7	ENSG00000127951	-5.092	0.005	DOWN
11	*IGHV3-30*	14	ENSG00000270550	-4.992	0.023	DOWN
12	*SH2D1B*	1	ENSG00000198574	-4.900	0.011	DOWN
13	*CYP27A1*	2	ENSG00000135929	-4.607	0.032	DOWN
14	*CD14*	5	ENSG00000170458	-4.417	0.005	DOWN
15	*VCAN*	5	ENSG00000038427	-4.227	0.005	DOWN
16	*IGKV4-1*	2	ENSG00000211598	-3.987	0.038	DOWN
17	*IGHA1*	14	ENSG00000211895	-3.896	0.016	DOWN
18	*IGHG1*	14	ENSG00000211896	-3.868	0.024	DOWN
19	*IGHG2*	14	ENSG00000211893	-3.681	0.027	DOWN
20	*PRF1*	10	ENSG00000180644	-3.397	0.011	DOWN
21	*IGLL5*	22	ENSG00000254709	-3.336	0.017	DOWN
22	*JCHAIN*	4	ENSG00000132465	-3.321	0.034	DOWN
23	*CYBB*	X	ENSG00000165168	-3.262	0.015	DOWN
24	*SPON2*	4	ENSG00000159674	-3.189	0.006	DOWN
25	*HK3*	5	ENSG00000160883	-3.166	0.011	DOWN
26	*HCK*	20	ENSG00000101336	-3.104	0.027	DOWN
27	*BLVRB*	19	ENSG00000090013	-3.046	0.039	DOWN
28	*CD163*	12	ENSG00000177575	-3.040	0.027	DOWN
29	*PDIA4*	7	ENSG00000155660	-3.010	0.015	DOWN
30	*FCGR3A*	1	ENSG00000203747	-2.951	0.020	DOWN
31	*IL1B*	2	ENSG00000125538	-2.934	0.022	DOWN
32	*IGKV3-20*	2	ENSG00000239951	-2.899	0.038	DOWN
33	*LILRA1*	19	ENSG00000104974	-2.890	0.027	DOWN
34	*SKAP2*	7	ENSG00000005020	-2.867	0.046	DOWN
35	*SPN*	16	ENSG00000197471	-2.818	0.024	DOWN
36	*ASGR2*	17	ENSG00000161944	-2.724	0.027	DOWN
37	*KIAA0930*	22	ENSG00000100364	-2.687	0.020	DOWN
38	*ARPC5*	1	ENSG00000162704	-2.686	0.015	DOWN
39	*SULF2*	20	ENSG00000196562	-2.664	0.023	DOWN
40	*PILRA*	7	ENSG00000085514	-2.659	0.025	DOWN
41	*CCL3*	17	ENSG00000277632	-2.652	0.017	DOWN
42	*FGR*	1	ENSG00000000938	-2.632	0.005	DOWN
43	*CAT*	11	ENSG00000121691	-2.616	0.023	DOWN
44	*LILRB1*	19	ENSG00000104972	-2.540	0.005	DOWN
45	*DOK3*	5	ENSG00000146094	-2.526	0.045	DOWN
46	*ANXA6*	5	ENSG00000197043	-2.514	0.035	DOWN
47	*PYCARD*	16	ENSG00000103490	-2.502	0.017	DOWN
48	*PECAM1*	17	ENSG00000261371	-2.480	0.023	DOWN
49	*SELPLG*	12	ENSG00000110876	-2.479	0.031	DOWN
50	*GZMB*	14	ENSG00000100453	-2.459	0.048	DOWN
51	*ADA2*	22	ENSG00000093072	-2.457	0.027	DOWN
52	*TALDO1*	11	ENSG00000177156	-2.436	0.038	DOWN
53	*TYMP*	22	ENSG00000025708	-2.424	0.015	DOWN
54	*GLIPR2*	9	ENSG00000122694	-2.405	0.041	DOWN
55	*GNS*	12	ENSG00000135677	-2.398	0.038	DOWN
56	*STAB1*	3	ENSG00000010327	-2.380	0.023	DOWN
57	*LILRB2*	19	ENSG00000131042	-2.377	0.027	DOWN
58	*JAML*	11	ENSG00000160593	-2.376	0.032	DOWN
59	*NCF2*	1	ENSG00000116701	-2.356	0.047	DOWN
60	*CTDSP1*	2	ENSG00000144579	-2.286	0.018	DOWN
61	*ITGAM*	16	ENSG00000169896	-2.237	0.038	DOWN
62	*RPS6KA1*	1	ENSG00000117676	-2.212	0.027	DOWN
63	*MIR23AHG*	19	ENSG00000267519	-2.176	0.017	DOWN
64	*UBXN11*	1	ENSG00000158062	-2.071	0.023	DOWN
65	*NEAT1*	11	ENSG00000245532	-1.962	0.022	DOWN
66	*AP2A1*	19	ENSG00000196961	-1.951	0.043	DOWN
67	*S1PR1*	1	ENSG00000170989	2.134	0.019	UP
68	*ICOS*	2	ENSG00000163600	2.157	0.024	UP
69	*CRYBG1*	6	ENSG00000112297	2.232	0.027	UP
70	*RPS28*	19	ENSG00000233927	3.808	0.032	UP

Genes with low expression (RPKM < 4) were also filtered.

Other genes among the top ten downregulated are associated with immune and inflammatory responses. This includes Fibrinogen-like protein 2 (FGL2), which is present in serum as a soluble protein and is a mediator of inflammation (29), and cytochrome b-245 beta chain (*CYBB). CYBB* encodes a subunit of the NADPH oxidase in phagocytes which is responsible for the microbicidal respiratory burst (30). *CYBB* is located in chromosome X and so may be related to the greater predisposition of females to Long COVID. Several Immunogloblin Light and Heavy Chain Variable (IGLV or IGHV) region genes were also present in the downregulated genes. All immunogloblin genes are located in chromosome fourteen, and as a group constituted the most frequently downregulated class of proteins in the Long COVID PBMCs. Seven out of the sixty-six downregulated genes belong to this class, namely *IGLV8-61*, *IGHV1-18*, *IGHV3-48*, *IGHV2-5*, *IGHV4-59*, *IGHV3-7*, and *IGHV3-30*. It is important to note that IGLV8-61 is a secretory protein and is predicted to be active in the extracellular space. Integrated proteomics data analyses, based on the publicly available proteomics data in ProteomicsDB, PaxDb, and MOPED showed that at the protein level, IGLV8-61 is mainly present in urine and skin (https://www.genecards.org/cgi-bin/carddisp.pl?gene=IGLV8-61). Secretory proteins in urine and/or blood are attractive biomarker candidates.

**Figure 4:**
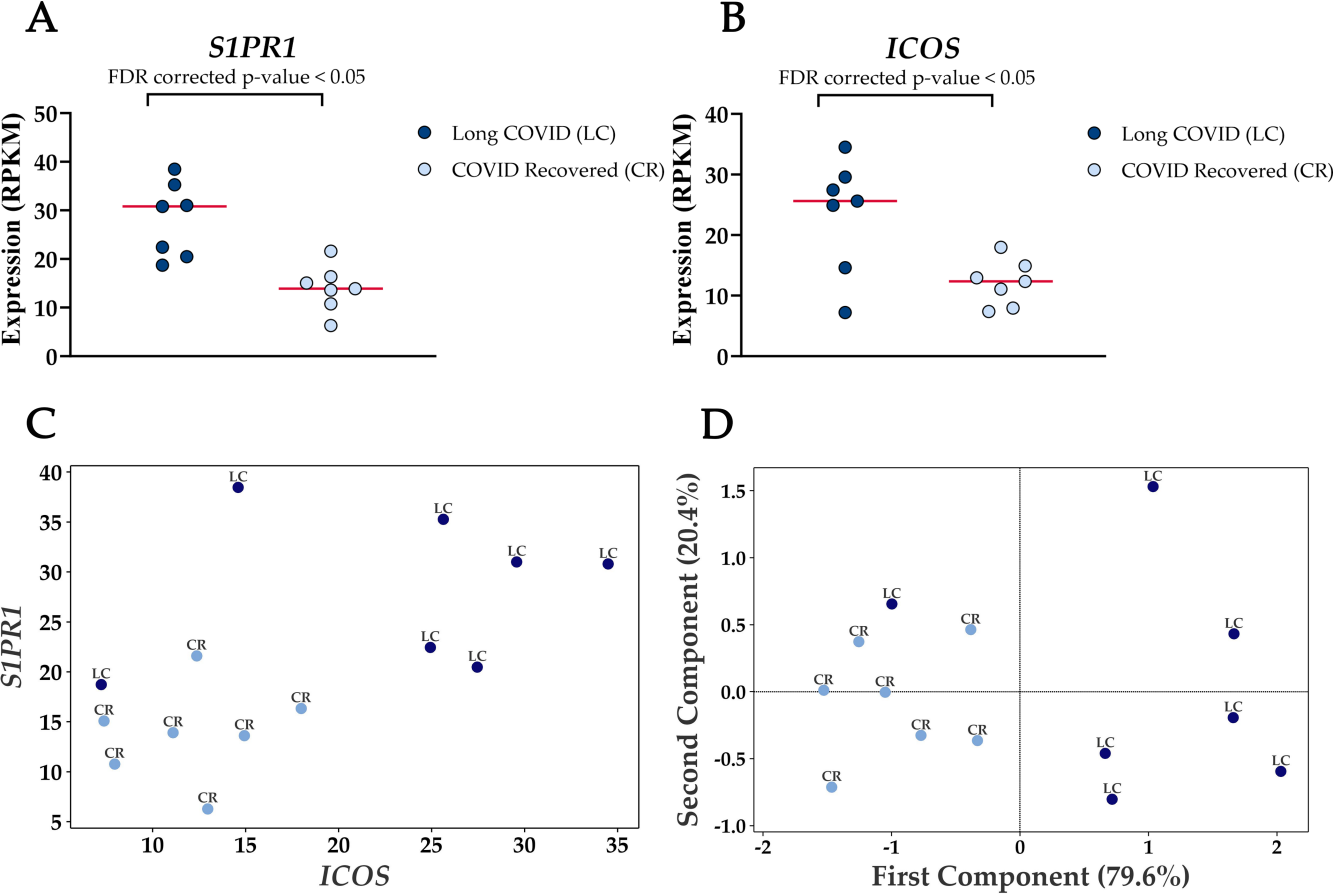
Elevated expression levels of *S1PR1* and *ICOS* in blood-derived PBMCs from Long COVID patients versus COVID recovered individuals. **(A)** Comparison of *S1PR1* expression between Long COVID and COVID recovered PBMCs. **(B)** Comparison of *ICOS* expression between Long COVID and COVID recovered PBMCs. **(C)** Scatter plot demonstrates the elevated expression of *S1PR1* and *ICOS* in Long COVID (LC) samples. **(D)** PCA analysis demonstrates that *S1PR1* and *ICOS* can discriminate all but one Long COVID (LC) sample from COVID recovered (CR), where the first component described 79.6% of variation in the expression data.

Four genes were significantly up-regulated in the Long COVID PBMC samples and were also associated with survival or immune pathways. Two such genes, Sphingolipid phosphate receptor 1 (*S1PR1*) and inducible co-stimulator (*ICOS*) (**Figure 4**) are associated with inflammation and the differentiation of T cells into T helper cells which produce inflammatory cytokines. Another, the 40S small ribosomal protein 28 (RPS28) has been proposed to play a role in the presentation of Major Histocompatibility Complex (MHC) class I peptides. The final up-regulated gene in Long COVID was crystallin beta-gamma domain containing 1 (CRYBG1) also known as Absent in Melanoma 1 (AIM1). This gene encodes an actin binding protein which acts as a tumour suppressor that is downregulated in various cancers (31).

Functional enrichment analysis was performed to further define the pathways that were differentially regulated in Long COVID PBMCs (**Table 3**). The “molecular function” Gene Ontology term was used to identify the molecular processes or activities enriched in the up- or down-regulated genes. Some of the molecular functions identified referred to very broad activities of signalling receptor binding, antigen binding and protein homodimerisation activity. However, the molecular functions with the highest strength were related to MHC class I molecules and immunoglobulin receptor activity. Immunoglobulin and MHC class I are essential for appropriate responses to infection and inflammation. Induction of the MHC class I pathway has been shown to be downregulated by SARS-CoV-2 infection (32) and the immunoglobulin receptor Fc receptor-like 2 has been identified as downregulated in several transcriptomic datasets from SARS-CoV-2 infected individuals (33).

**Table 3:** Enrichment analysis of genes that were up- or down-regulated in Long COVID compared to COVID recovered individuals in terms of molecular function.

“Molecular function” (STRING)	STRING pathway strength	pFDR	Proteins
inhibitory receptor binding to MHC class I	2.02	0.0099	LILRA1,LILRB1,LILRB2
Immunoglobulin receptor binding	1.82	0.0166	FGR,IGLL5,JCHAIN
MHC class I protein binding	1.74	0.0225	PILRA, LILRB1, LILRB2
Antigen binding	1.3	0.0374	LILRA1, IGLL5, JCHAIN, SPON2
Glycosaminoglycan binding	0.94	0.0414	GNS, VCAN, STAB1, ANXA6, SULF2, ADA2
Protein homodimerisation activity	0.77	0.0016	CAT, PYCARD, CSF1R, RBPMS2, LILRB1, JAML, GLIPR2, LILRB2, TYMP, ADA2, JCHAIN, PECAM1
Signalling receptor binding	0.63	0.00049	PILRA, SELPLG, PYCARD, FGL2, IL1B, S1PR1, LILRB1, JAML, AP2A1, FGR, HCK, LILRB2, TYMP, ADA2, IGLL5, JCHAIN, CCL3, ITGAM

The other molecular function which was enriched in the up- or down-regulated transcripts was glycosaminoglycan binding. Glycosaminoglycans (GAGs) are ubiquitously and abundantly expressed on the surface of cells or in the extracellular matrix and interact with many proteins including chemokines, cytokines and growth factors. Due to the diversity of these interactions, GAGs play important roles in countless biological functions including cell growth and proliferation, resistance to invasion by pathogens and migration of immune cells (34).

### 3.4 Reduced expression levels of LILRB1 and LILRB2 can effectively differentiate long COVID blood samples from those of recovered individuals

**Figure 5:**
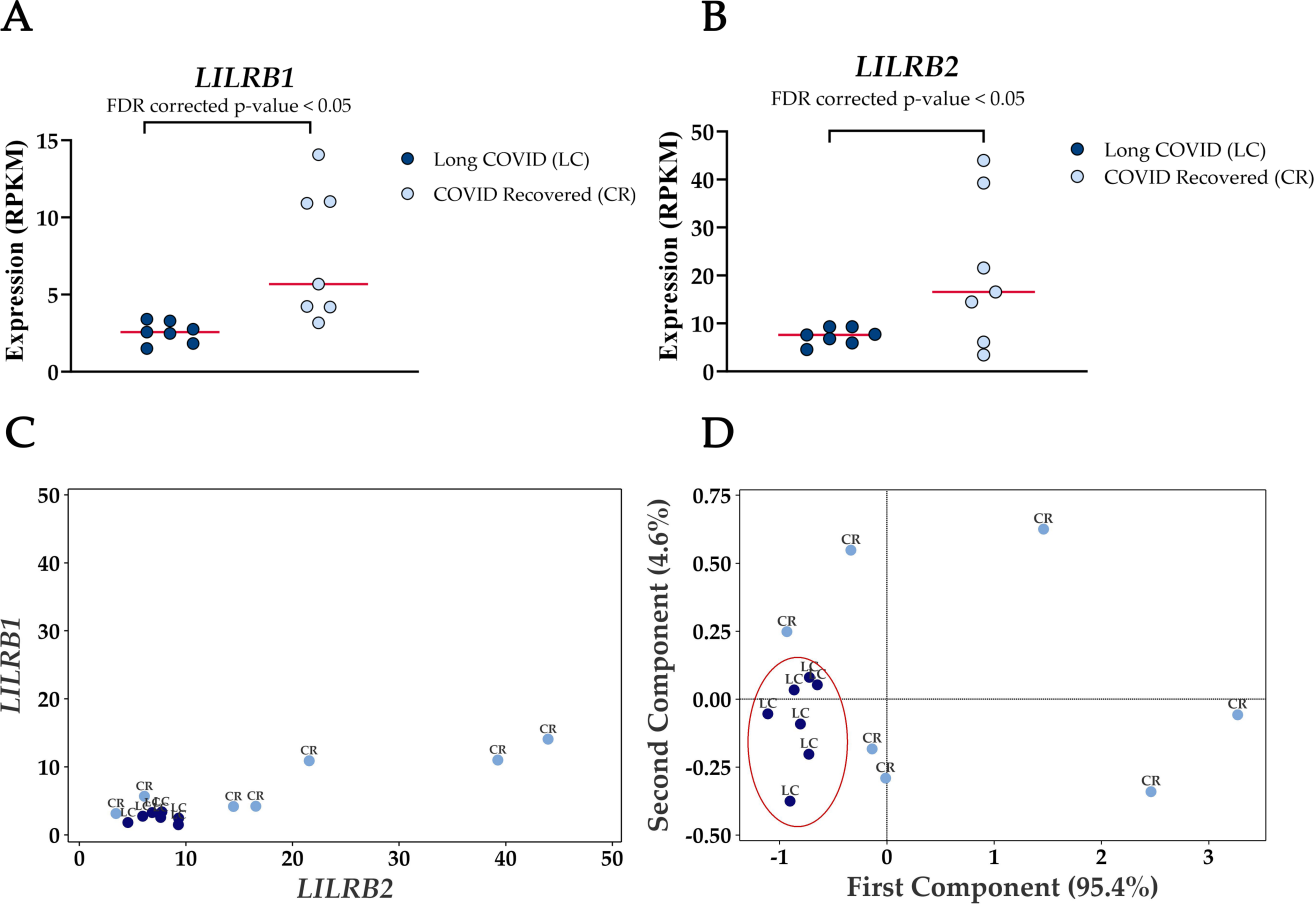
Reduced expression levels of *LILRB1* and *LILRB2* in blood-derived PBMCs effectively differentiate Long COVID patients from COVID recovered individuals. **(A)** Comparison of *LILRB1* expression between Long COVID and COVID recovered PBMCs. **(B)** Comparison of *LILRB2* expression between Long COVID and COVID recovered PBMCs. **(C)** Scatter plot demonstrates the reduced expression of *LILRB1* and *LILRB2* in Long COVID (LC) samples. **(D)** PCA analysis demonstrates the power of *LILRB1* and *LILRB2* to discriminate Long COVID (LC) samples from COVID recovered (CR) where the first component described 95.4% of variation in the expression data.

The most frequently occurring genes in the identified molecular functions were leukocyte immunoglobulin-like receptor subfamily B member 1 (*LILRB1*) and leukocyte immunoglobulin-like receptor subfamily B member 2 (*LILRB2*) (**Table 3**) and both genes were significantly downregulated in Long COVID (**Figure 5**). *LILRB1* and *LILRB2* are frequently expressed in immune cells and function largely to regulate antigen presenting cells such as macrophages, dendritic cells and B cells, thus are important in a variety of innate and adaptive immune responses (35, 36). Specifically, the gene products are involved in inhibitory responses which suppress downstream pathways to mediate immunosuppression. Downregulation of *LILRB1* and *LILRB2* as observed here in the Long COVID PBMCs is therefore indicative of a heightened immune response and inflammation.

PCA analysis demonstrates the power of *LILRB1* and *LILRB2* in completely discriminating Long COVID from COVID recovered PBMCs. The first component described 95.4% of variation in the expression data. The ability of only two genes to distinguish the two cohorts with no overlap is a promising avenue for future validation as a prospective biomarker.

## 4 Discussion

Utilizing PBMC transcriptomic profiling in a small cohort, this study has revealed information about the molecular mechanisms of Long COVID and has identified a prospective transcriptomic signature of the disease in PBMCs for subsequent validation.

Analysis of PBMC transcriptomes from Long COVID individuals compared to COVID recovered controls revealed that the vast majority of differentially expressed genes were downregulated in Long COVID. The most frequently downregulated family of genes in Long COVID PBMCs was Immunoglobulin Heavy Chain Variable Region (IGHV), constituting six of the top ten downregulated genes. This observation in Long COVID samples contrasts with the up-regulation of this family of genes reported in acute SARS-CoV-2 infection (33). An analysis of transcriptomic data from nine different studies that compared SARS-CoV-2 infected cohorts to either healthy controls or other respiratory diseases identified that the expression of IGHV genes was most consistently up-regulated specifically in severe SARS-CoV-2 infections (33). This is noteworthy since severe infections are the most associated with development of Long COVID. A shift from elevated IGHV expression during the acute phase to reduced IGHV expression in Long COVID suggests dysregulation, and perhaps overactivity of the regulatory pathways involved in bringing the immune response back down over time. Interestingly, all of these genes are located in chromosome fourteen which suggests a possible hotspot of shared regulatory control that should be investigated in future studies.

Induction of cell death pathways to decrease the numbers of activated immune cells is an important homeostatic mechanism which can backfire during severe viral infection. This depletion of lymphocytes is termed lymphopenia. The mechanisms of cell death underpinning lymphopenia are diverse. T cell lymphopenia is a feature of many respiratory viral infections and is common in SARS-CoV-2 infection, particularly severe patients (37). T cell lymphopenia has been reported to be more severe in COVID patients and takes longer to resolve than in other viral infections (38). In the current study we did observe a decrease in the expression of genes associated with cell survival. While not measured directly, we also observed that the Long COVID PBMC samples died more quickly than the COVID recovered controls once recovered from frozen storage. Together this is unsurprising given that lymphopenia has been reported to be common in Long COVID (39–41). What these observations do suggest is that the mechanism by which lymphopenia occurs in Long COVID may involve the downregulation of genes associated with cell survival that we observed.

In our data we observed a significant increase in expression of the inducible T cell co-stimulator *ICOS*. *ICOS* promotes all fundamental T cell responses to foreign antigen and is crucial in mediating inflammation (42, 43). Among many other functions, *ICOS* enhances differentiation of T cells into T helper cells which produce pro-inflammatory cytokines. Pro-inflammatory cytokines and persistent low-grade inflammation are ubiquitously reported in Long COVID, although elevated levels of pro-inflammatory cytokines have been reported in recovered individuals too (44). Our observation of elevated *ICOS* expression, on average six months after infection, confirms that a pro-inflammatory state exists in Long COVID PBMCs until at least six months and that *ICOS* levels do intuitively wane over time in COVID recovered individuals, as would also be expected of the levels of pro-inflammatory cytokines.

The lipid mediator sphingolipid phosphate (S1P) and the sphingolipid phosphate receptors (S1PR) play critical roles in immune responses. S1PR1 is highly expressed on immune cells and together with S1P has been implicated as a regulator of inflammatory diseases. The S1P/S1PR1 pathway is essential for the trafficking of immune cells and in the differentiation of T helper cells (45). *S1PR1* is up-regulated in several autoimmune diseases such as multiple sclerosis, systemic lupus erythematosus or rheumatoid arthritis and agents which inhibit S1PR1 are being investigated for their therapeutic potential (46). It is proposed that, in autoimmune conditions, increased S1PR1 inhibits the number and functions of regulatory T cells leading to increased production of inflammatory cytokines. Elevated *ICOS* expression is also associated with autoimmune diseases such as rheumatoid arthritis and lupus nephritis (47). Thus, the concomitant up-regulation of *ICOS* and *S1PR1* in our Long COVID PBMCs likely forms a regulatory axis underpinning persistent inflammation and could be related to an autoimmune component based on our knowledge of other diseases.

Regulation of an appropriate inflammatory response is largely dependent on the expression and activation of immunoregulatory receptors, of which the leukocyte immunoglobulin like receptors (LILRBs) are key players. These receptors regulate inflammation via the control of many cellular processes including cell survival, phagocytosis, cell migration, cytokine production and cell death (48). In our data we observed a large downregulation of two LILRBs, *LILRB1* and *LILRB2*, both of which inhibit inflammatory processes. Reduced expression or impaired function of LILRB1 and LILRB2 have been associated with inflammatory autoimmune conditions such as rheumatoid or psoriatic arthritis and systemic lupus erythematosus (49–52). This supports the potential role of reduced expression in promoting a sustained inflammatory state in Long COVID. Future studies characterizing *LILRB1* and *LILRB2* expression and function in SARS-CoV-2 infection and over time would be useful in determining how these receptors contribute to the severity of disease and susceptibility to development of Long COVID.

In this study we showed that the reduced expression of *LILRB1* and *LILRB2* alone discriminated a small pilot cohort of Long COVID PBMC samples from COVID recovered controls without overlap. This and the biological roles of these two genes being potentially related to the underlying pathology emphasize their potential as blood-based biomarkers of Long COVID. Given the small sample size of this pilot study this requires validation in larger cohorts, across different disease durations and in different disease groups.

## Data Availability

The data presented in the study are deposited in the Sequence Read Archive NCBI repository, accession number PRJNA1184005.
